# Molecular characterization analysis of PANoptosis subtyping and identification of elastin as a novel therapeutic target in colorectal cancer

**DOI:** 10.1016/j.gendis.2025.101560

**Published:** 2025-02-18

**Authors:** Yongmin Li, Dongxu Liu, Yiming Ma, Yu Liang, Yupeng Ren, Qingkai Meng, Bin Ma

**Affiliations:** a Department of Colorectal Surgery, Cancer Hospital of China Medical University, Cancer Hospital of Dalian University of Technology, Liaoning Cancer Hospital & Institute, Shenyang, Liaoning 110042, China; b Department of Medical Oncology, Cancer Hospital of China Medical University, Cancer Hospital of Dalian University of Technology, Liaoning Cancer Hospital & Institute, Shenyang, Liaoning 110042, China

PANoptosis is a novel mode of programmed cell death and plays an important role in tumor development and metastasis.^1^^,^^2^ Numerous studies have shown that PANoptosis plays a crucial role in cancer,^3^, ^4^, ^5^ however, there is limited research to reveal the association between PANoptosis and colorectal cancer (CRC). Based on our results, a PANoptosis-related signature (PRS) was developed by a combination of 101 machine learning algorithms that could accurately predict survival outcomes in TCGA and four external validation cohorts exhibited high performance when compared with other public signatures. The PRS was highly correlated with tumor microenvironment, immunotherapy, drug sensitivity, and genomic mutation. Moreover, six and nine candidate drugs from CTRP and PRISM databases respectively were identified for the high PRS score patients. A candidate therapeutic target, elastin (ELN), was screened in CRC cell lines, and its expression and clinical value were further evaluated in the GSE132257 dataset. Furthermore, the clinical significance of ELN was evaluated through CRC tissue samples. In summary, the present study uncovered a promising application of the signature in the prognosis of CRC. The potential therapeutic target of ELN might provide direction and tailor more effective treatment for CRC.

Firstly, based on the transcriptome profile of PANoptosis regulators, patients were classified into two molecular subtypes through consensus clustering analysis, named C1 and C2 (Fig. 1A; Fig. S1A–D). To further elucidate the potential molecular mechanism of subtypes, we conducted a differential expression analysis between two molecular subtypes (Fig. S1E). Before PRS construction, we extracted the common differentially expressed genes from five datasets and performed univariate Cox regression analysis in each dataset. The common risky or protective genes in at least three cohorts were retained and a total of 124 genes were subjected to the machine learning analysis. Based on the average C-index value, the Enet algorithm with *α* = 0.1 was determined to be the optimal prognostic signature, with an average C-index value of 0.78 (Fig. 1B). The CRC patients were then classified into high- and low-risk groups based on the median PRS score across all cohorts. The CRC patients in the high PRS score group showed a significantly poor overall survival in TCGA (Fig. 1C), GSE38832, GSE29612, GSE17537, and GSE29612 cohorts (Fig. S2A–D). The receiver operating characteristic curve analysis result demonstrated that the PRS showed a good performance at the 1-, 3-, and 5-year overall survival across TCGA (Fig. 1D) and other CRC cohorts (Fig. S2A–D). Moreover, we systematically collected 32 recently published signatures for CRC and compared them with our machine-learning signature. Notably, the PRS demonstrated superior C-index performance in TCGA, GSE17536, GSE17537, GSE29612, and GSE38832 cohorts than almost all public signatures (Fig. S3).

**Figure 1:**
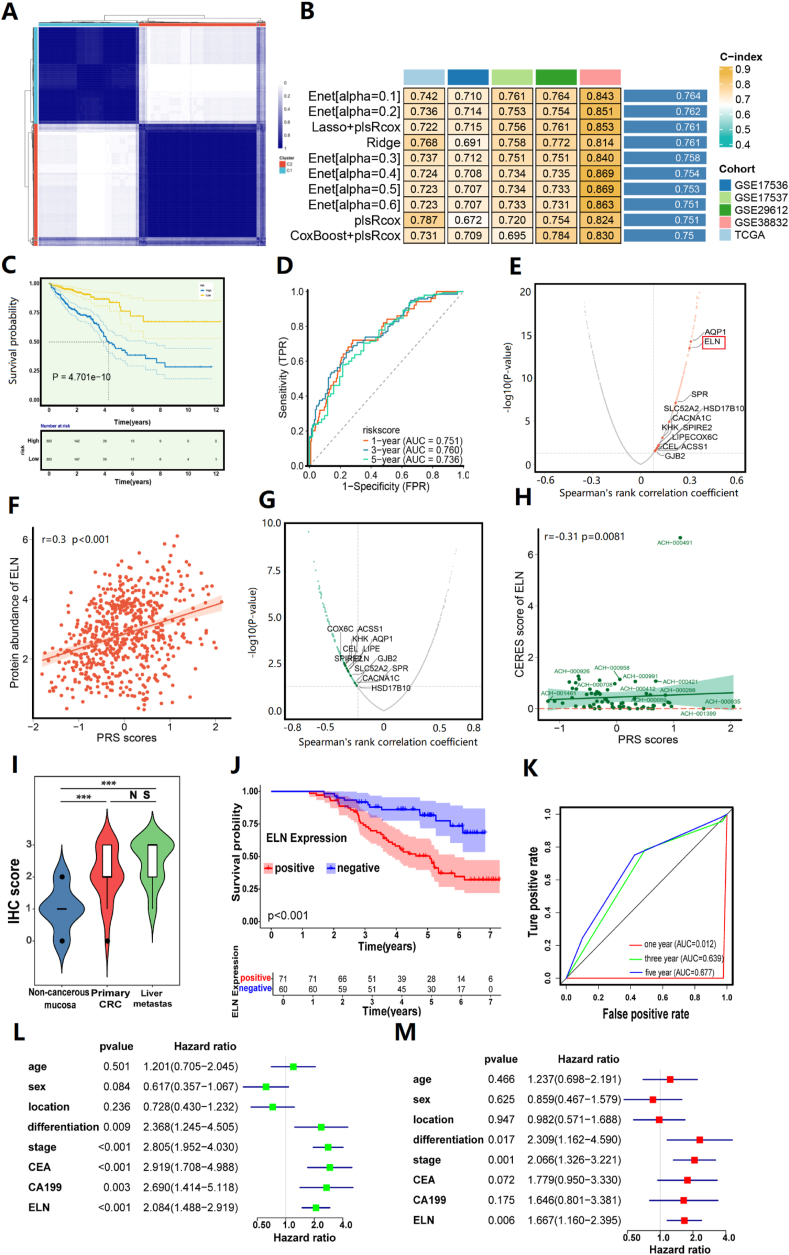
Molecular characterization analysis of PANoptosis subtyping and identification of elastin (ELN) as a novel therapeutic target in colorectal cancer (CRC). **(A)** Identification of molecular subtypes based on PANoptosis regulator expression. Principal component analysis was performed to classify patients into C1 and C2 subtypes. **(B)** Construction and evaluation of PANoptosis-related signature (PRS) in TCGA cohorts. The PRS was developed based on the combination of 101 machine learning algorithms. The C-index value was calculated in the TCGA and GEO cohorts (the top 10 C-index were shown). **(C, D)** Kaplan–Meier curves and receive operator curves were used for survival prediction and prognostic value evaluation of PRS in TCGA. **(E, F)** Characterization of PRS-related target. Volcano plot (left) and scatter plots (right) display Pearson’s correlation coefficient and significance between PRS scores and expression of drug targets in the TCGA cohort. The red dot represents the samples of TCGA. **(G, H)** Volcano plot (left) and scatter plots (right) display Pearson’s correlation coefficient and significance between PRS scores and CERES scores of drug targets in the TCGA cohort. The green dot represents each CRC cell line. **(I)** Comparison of ELN expression in noncancerous mucosa, paired primary CRC, and liver metastases by immunohistochemistry. **(J)** Kaplan–Meier plots of CRC specimens with negative and positive ELN expression. **(K)** Time-dependent receiver operating characteristic analysis of ELN showed the overall survival of patients with CRC. **(L)** Univariate Cox regression of ELN expression and clinical parameters in CRC. **(M)** Multivariate Cox regression of ELN expression and clinical parameters in CRC. ∗∗∗*P* < 0.001; ns, no significance.

To further demonstrate the potential of PRS as an independent prognostic factor, we conducted univariate and multivariate Cox regression analysis on the clinical factors and PRS score in the five cohorts. As the result showed, PRS was identified as an independent factor across the five datasets (Fig. S4A–E). In addition, the occurrence of tumors is caused by a series of mutations or abnormalities accumulating at the genomic level. Therefore, we compared the gene variations between the high- and low-PRS groups in the TCGA cohort. We selected the top 30 most mutated genes and visualized them in the waterfall plot. The mutation rates of most genes, including TP53, MAGEC1, ROS1, BAI3, DCAF4L2, and MYO16, were observed to be significantly higher in the high PRS group compared with the low PRS group (Fig. S5A). Moreover, to comprehensively investigate the impact of PRS on the level of immune cell infiltration in the CRC microenvironment, we utilized distinct seven immune infiltration algorithms to assess the infiltration levels of different types of immune cells in CRC and then compared the differences between the high and the low PRS group. The result indicated that most of the immune cells calculated by the seven algorithms were negatively correlated with PRS scores, such as CD4^+^ T cell, CD8^+^ T cell, B cell, and natural killer cell, while the infiltration level of endothelial cell, M1 macrophage, M0 macrophage, and cancer fibroblast was positively correlated with PRS score (Fig. S5B, C).

To identify the potential drug for the treatment of high PRS group patients with poor survival, we retrieved a previously reported 6125 drug information and carried out a multi-step analysis to identify candidate drug targets. Firstly, we performed Pearson correlation analysis between the drug target expression and PRS score, and 457 potential targets were identified (Pearson *r* > 0 and *P* < 0.05) (Fig. 1E, F). Next, Pearson correlation analysis was also performed between CERES score and PRS score based on CRC cell lines, and 182 candidate drug targets were screened (Pearson *r* < 0 and *P* < 0.05) (Fig. 1G, H). As a result, 13 drug targets including GJB2, AQP1, ACSS1, CACNA1C, LIPE, COX6C, HSD17B10, KHK, SPIRE2, SLC52A2, SPR, ELN, and CEL were screened. Among these targets, the CERES score of ELN in most CRC cell lines was close to zero, suggesting that ELN might be crucial for CRC, and blocking its function in high PRS group patients could lead to a favorable treatment outcome. Moreover, we also performed Pearson correlation analysis between the drug’s area under curve value and ELN gene expression based on cell lines, and 19 drugs were identified as correlated with ELN (|Pearson *r*| > 0.3 and *P* < 0.05) (Fig. S6A). Among these drugs, erlotinib was most positively correlated with ELN, and the mechanism of action of drugs revealed that erlotinib was involved in the EGFR signaling pathway (Fig. S6B). Additionally, we further evaluated the expression level of ELN on the single-cell level. Interestingly, CD4^+^ T cells were the predominant cells in the low PRS group versus the high PRS group in tumor and normal tissue, while plasma cells mainly focused on the high PRS group in tumor and normal tissue (Fig. S7A–D). We then evaluated the level of PRS score and ELN on the nine cells and discovered that PRS score was mainly expressed in macrophages and fibroblasts, while ELN was only expressed in fibroblasts (Fig. S7E, F).

Finally, immunohistochemistry analysis of ELN was conducted in our cohort CRC tissue samples. The results demonstrated a significant overexpression of ELN in both primary CRC tissues and liver metastasis tissues (Fig. 1I). In addition, a Kaplan–Meier curve was constructed to evaluate the prognostic significance of ELN in a cohort of 131 CRC patients. The analysis revealed that patients with high ELN expression exhibited significantly inferior overall survival compared with those with low ELN expression (Fig. 1J). The area under curve values for ELN expression indicated that ELN accurately discriminated between the high- and low-expression groups (Fig. 1K). Both univariate and multivariate Cox regression analyses confirmed ELN as a robust prognostic marker, with statistical significance observed in both analyses (Fig. 1L, M). Furthermore, an investigation into the association between ELN expression and clinicopathological characteristics of the 131 CRC patients was undertaken (Table S1).

In conclusion, we established a robust PRS based on the combination of 101 machine-learning algorithms. Our results suggest that PRS has a promising clinical application value and ELN might be a novel therapeutic target in CRC.

## CRediT authorship contribution statement

**Yongmin Li:** Writing – original draft, Validation, Conceptualization. **Dongxu Liu:** Visualization, Software, Investigation, Formal analysis. **Yiming Ma:** Software, Resources, Methodology, Formal analysis, Data curation. **Yu Liang:** Visualization, Software, Resources, Funding acquisition, Formal analysis, Data curation. **Yupeng Ren:** Writing – review & editing, Visualization, Validation. **Qingkai Meng:** Writing – review & editing, Visualization, Validation, Conceptualization. **Bin Ma:** Writing – review & editing, Writing – original draft, Validation, Funding acquisition, Formal analysis, Data curation, Conceptualization.

## Ethics declaration

The research involving human participants was approved by the Human Ethics Committees of the Liaoning Cancer Hospital (No. 20210804 GP). The patients/participants provided their written informed consent to participate in the study.

## Data availability

The datasets used and/or analyzed during this study are available from the corresponding author upon reasonable request.

## Funding

This work was supported by the Cultivation Program of National Science Foundation of Liaoning Cancer Hospital (No. 2021-ZLLH-03), the Medical and Industrial Cross Joint Fund of Dalian University of Technology-Liaoning Cancer Hospital (No. LD2023027), the Liaoning Province Science and Technology Plan Joint Program (Applied Basic Research Project) (China) (No. 2023JH2/101700150), and the 10.13039/501100001809National Natural Science Foundation of China (No. 82303131).

## Conflict of interests

The authors declared no conflict of interests.
