# Supplementary material for: Molecular characterization analysis of PANoptosis subtyping and identification of elastin as a novel therapeutic target in colorectal cancer
Source: Genes Dis. 2025 Feb 18;13(1):101560. doi: 10.1016/j.gendis.2025.101560 (PMC12624590; doi:10.1016/j.gendis.2025.101560)
Supplement: Multimedia component 1 [file mmc1.docx]

**Supplementary Materials**

**Materials and Methods**

**Data acquisition and pre-processing**

Five datasets including GSE17536, GSE17537, GSE29612, GSE38832 and TCGA-COADREAD were retrieved from Gene Expression Omnibus (GEO) and The Cancer Genome Atlas Program (tcga, https://portal.gdc.cancer.gov/), respectively. For the TCGA-COADREAD dataset, we firstly downloaded the TCGA-COAD and TCGA-READ raw reads count data and transform to transcripts per million (tpm) value. Batch effects between TCGA-COAD and TCGA-READ dataset were then removed using the “sva” R package ^1^. The corresponding genomic mutation data and clinical information were also obtained. In addition, 66 PANoptosis-related regulators were retrieved from previously reports ^2^.

**Molecular subtypes analysis**

According to the PANoptosis regulators RNA expression level, patients from TCGA dataset were clustered using the “ConsensusClusterPlus” R package to identify PANoptosis-related molecular expression pattern ^3^. The optimal cluster number was determined by cumulative distribution function curve and delta curve. In addition, principal component analysis (PCA) was utilized to reduce dimensionality to identify the reliability of clustering number. Differentially expressed genes (DEGs) were identified between molecular subtypes using “limma” R package based on the cutoff: “adjusted P < 0.05” ^4^. Pathway enrichment analysis was conducted using the “clusterProfiler” R package based on Kyoto Encyclopedia of Genes and Genomes (KEGG) database ^5^.

**Machine-learning analysis**

Before running machine learning analysis, we firstly extracted the DEGs shared by the five datasets, and then conducted univariate cox regression analysis in each dataset, retaining genes that were identified as either risky or protective in at least 3 datasets. Subsequently, we integrated 101 algorithm combinations from 10 algorithms based on 10-fold cross-validation to develop a stable and reliable PANoptosis-related signature (PRS). The 10 algorithms including CoxBoost, elastic network (Enet), generalized boosted regression modeling (GBM), Lasso, partial least squares regression for Cox (plsRcox), Ridge, random survival forest (RSF), stepwise cox, supervised principal components [SuperPC] and survival support vector machine (survival-SVM). The Harrell’s concordance index (C-index) value across five datasets were subsequently calculated, and an PRS with high average C-index value was selected as the optimal risk model.

**Quantification of immune cells**

Seven immune infiltration algorithms including xCell, TIMER, MCPcounter, ESTIMATE, EPIC, CIBERSORT and CIBERSORT-ABS were applied to quantify immune cells infiltration, and implement by “IOBR” R package ^6^.

**Gene set variation analysis**

Gene set variation analysis (GSVA) is a non-parametric, unsupervised algorithm ^7^. In contrast to Gene set enrichment analysis (GSEA), GSVA can calculate the enrichment score of a specific gene set in each ccRCC sample. The signature of c2.cp.kegg_legacy.v2023.2.Hs.symbols obtained from MSigDB database was served as reference gene set. Significant enriched pathways were screened based the cutoff: adjusted value < 0.05.

**Prediction of drugs and therapeutic target**

The drug sensitivity data for CRC cell lines were sourced from the CTRP and PRISM database, available at https://portals.broadinstitute.org/ctrp and <https://depmap.org/portal/prism/,> respectively. In both databases, the AUC values for each drug have been calculated, and the missing AUC values were imputed via the k-nearest neighbor (KNN) algorithm. Due to the CRC cell lines all sourced from cancer cell line encyclopedia (CCLE, https://sites.broadinstitute.org/ccle/) database, the expression profile data in CCLE were then subjected to CTRP and PRISM analysis. In addition, the CERES scores were obtained from CRISPR knockout screens of 18,333 genes in 739 cell lines from the Dependency Map (DepMap) portal (https://depmap.org/portal/). These scores can be used to measure the dependency of specific genes of interest in a cell line. A lower CERES score indicates a higher likelihood that the gene is essential for cell growth and survival in that cell line.

**Single-cell RNAseq analysis**

The single-cell RNAseq dataset GSE132257 comprised of 5 CRC tissue and 5 match normal tissue were retrieved form GEO database ^8^. We then applied the Seurat R package to the downstream analysis. The cells were retainedbased on the following criterion: i. minimum number of cells is at least 3; ii. less than 10% mitochondria-related genes expressed. iii. more than 200 but less than 2,500 genes expressed. Next, we will utilize the “Harmony” R package to integrate batches and apply tSNE and UMAP algorithms for dimensionality reduction analysis and visualization of genes ^9^. The cells were annotated using known cell makers from previously studies and “SingleR” package.

**Immunohistochemistry**

IHC analysis was conducted on the samples to quantify the expression levels of ELN. Specifically, ELN antibodies sourced from HUABIO-China (Catalog#: ER1908-02), were employed to incubate with tissue sections. Subsequently, the assessment of ELN expression involved counting the percentage of positively stained immunoreactive cells and evaluating the intensity of the staining. The IHC staining outcomes for ELN were graded on a scale ranging from “−” to “+++”, where “−” to “++” represented negative staining and “+” to “+++” indicated varying degrees of positive staining. This grading process was carried out by two impartial and experienced pathologists, who were blinded to the identity of the samples to ensure objectivity in the analysis.

**Statistically analysis**

All analysis was implemented in the R environment with the version 4.2.3. Differences between two groups were compared using Wilcoxon rank test or T-test. Correlation analysis is conducted using the Pearson coefficient test. The relationship between clinical and PRS is assessed using the chi-square test. Receive operator curve (ROC) analysis was used to evaluate the prognostic value of PRM in the five cohorts. Kaplan-Meier curve and log-rank test was applied to estimate the survival difference between high- and low-risk groups. The independence of PRM score was validated through univariate and multivariate cox regression analysis. For all analyses, p < 0.05 was considered significant.

**References for Methods and Materials**

1. Leek, J.; Johnson, W.; Parker, H.; Jaffe, A.; Storey, J., The SVA package for removing batch effects and other unwanted variation in high-throughput experiments. *Bioinformatics (Oxford, England)* **2012,** *28*, 882-3.

2. Wang, X.; Sun, R.; Chan, S.; Meng, L.; Xu, Y.; Zuo, X.; Wang, Z.; Hu, X.; Han, Q.; Dai, L.; Bai, T.; Yu, Z.; Wang, M.; Yang, W.; Zhang, H.-B.; Chen, W., PANoptosis-based molecular clustering and prognostic signature predicts patient survival and immune landscape in colon cancer. *Frontiers in genetics* **2022,** *13*.

3. Wilkerson, M.; Hayes, D., ConsensusClusterPlus: A class discovery tool with confidence assessments and item tracking. *Bioinformatics (Oxford, England)* **2010,** *26*, 1572-3.

4 Ritchie, M.; Phipson, B.; Wu, D.; Hu, Y.; Law, C.; Shi, W.; Smyth, G., LIMMA powers differential expression analyses for RNA-sequencing and microarray studies. *Nucleic acids research* **2015,** *43*.

5. Wu, T.; Hu, E.; Xu, S.; Chen, M.; Guo, P.; Dai, Z.; Feng, T.; Zhou, L.; Tang, W.; Zhan, L.; Fu, X.; Liu, S.; Bo, X.; Yu, G., clusterProfiler 4.0: A universal enrichment tool for interpreting omics data. *The Innovation* **2021,** *2*, 100141.

6. Zeng, D.; Ye, Z.; Shen, R.; Yu, G.; Wu, J.; Xiong, Y.; Zhou, R.; Qiu, W.; Huang, N.; Sun, L.; Li, X.; Bin, J.; Liao, Y.; Shi, M.; Liao, W., IOBR: Multi-Omics Immuno-Oncology Biological Research to Decode Tumor Microenvironment and Signatures. *Frontiers in Immunology* **2021,** *12*, 687975.

7. Hänzelmann, S.; Castelo, R.; Guinney, J., GSVA: Gene set variation analysis for microarray and RNA-Seq data. *BMC bioinformatics* **2013,** *14*, 7.

8. Lee, H.-O.; Hong, Y.; Etlioglu, H.; Cho, Y. B.; Pomella, V.; Bosch, B.; Vanhecke, J.; Verbandt, S.; Hong, H.; Min, J.-W.; Kim, N.; Eum, H.; Qian, J.; Boeckx, B.; Lambrechts, D.; Tsantoulis, P.; De Hertogh, G.; Chung, W.; Lee, T.; Park, W.-Y., Lineage-dependent gene expression programs influence the immune landscape of colorectal cancer. *Nature Genetics* **2020,** *52*, 1-10.

9. Korsunsky, I.; Millard, N.; Fan, J.; Slowikowski, K.; Zhang, F.; Wei, K.; Baglaenko, Y.; Brenner, M.; Loh, P.-r.; Raychaudhuri, S., Fast, sensitive and accurate integration of single-cell data with Harmony. *Nature Methods* **2019,** *16*, 1-8.


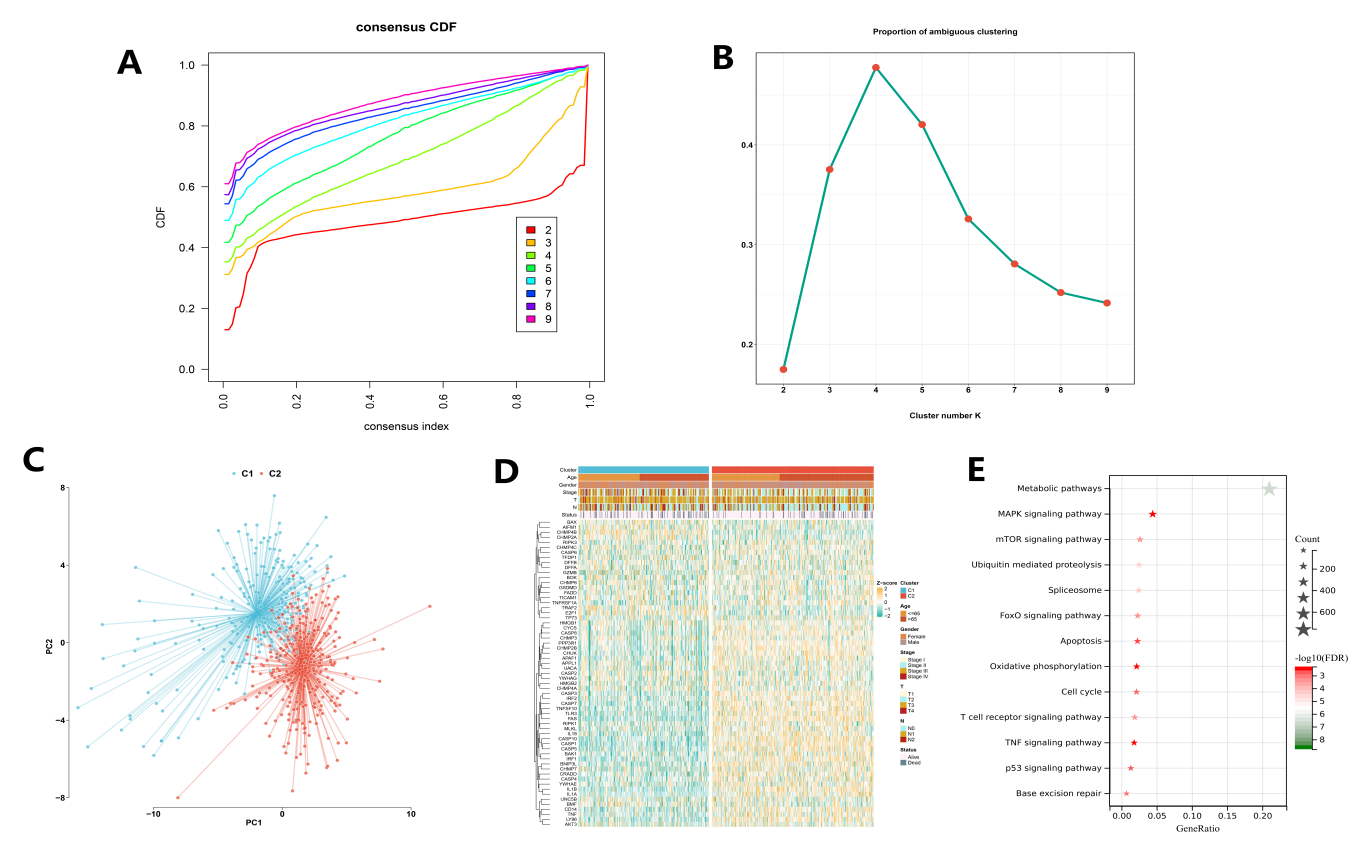


**Fig. S1** Identification of molecular subtypes based on PANoptosis regulator expression. (A) CDF curve of consensus matrix from k=2 to k=9, each color line represents a cluster number. (B) PAC curve of consensus matrix from k= 2 to k=9. (C) PCA analysis was performed to classify patients into C1 and C2 subtypes. (D) The expression profile of PANoptosis regulators in two molecular subtypes, (E) Kaplan-Meier curves analysis to evaluate the survival difference between two subtypes.


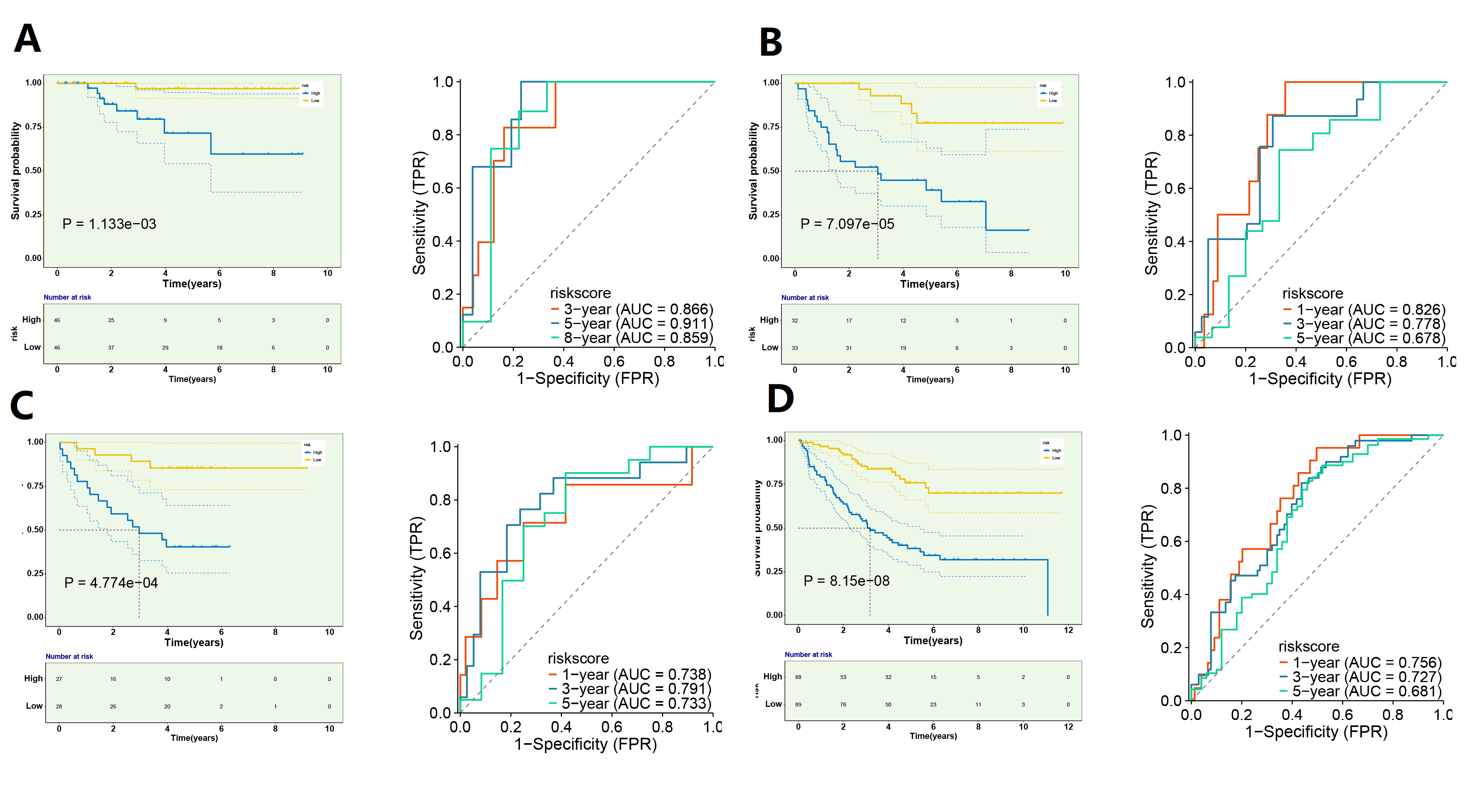


**Fig. S2** Construction and evaluation of PRS in multiple GEO cohorts. (A-D) Kaplan-Meier curves and receive operator curves were used for survival prediction and prognostic value evaluation of PRS in GSE38832 (A), GSE29612 (B), GSE17537 (C) and GSE29612 (D) cohorts, respectively.


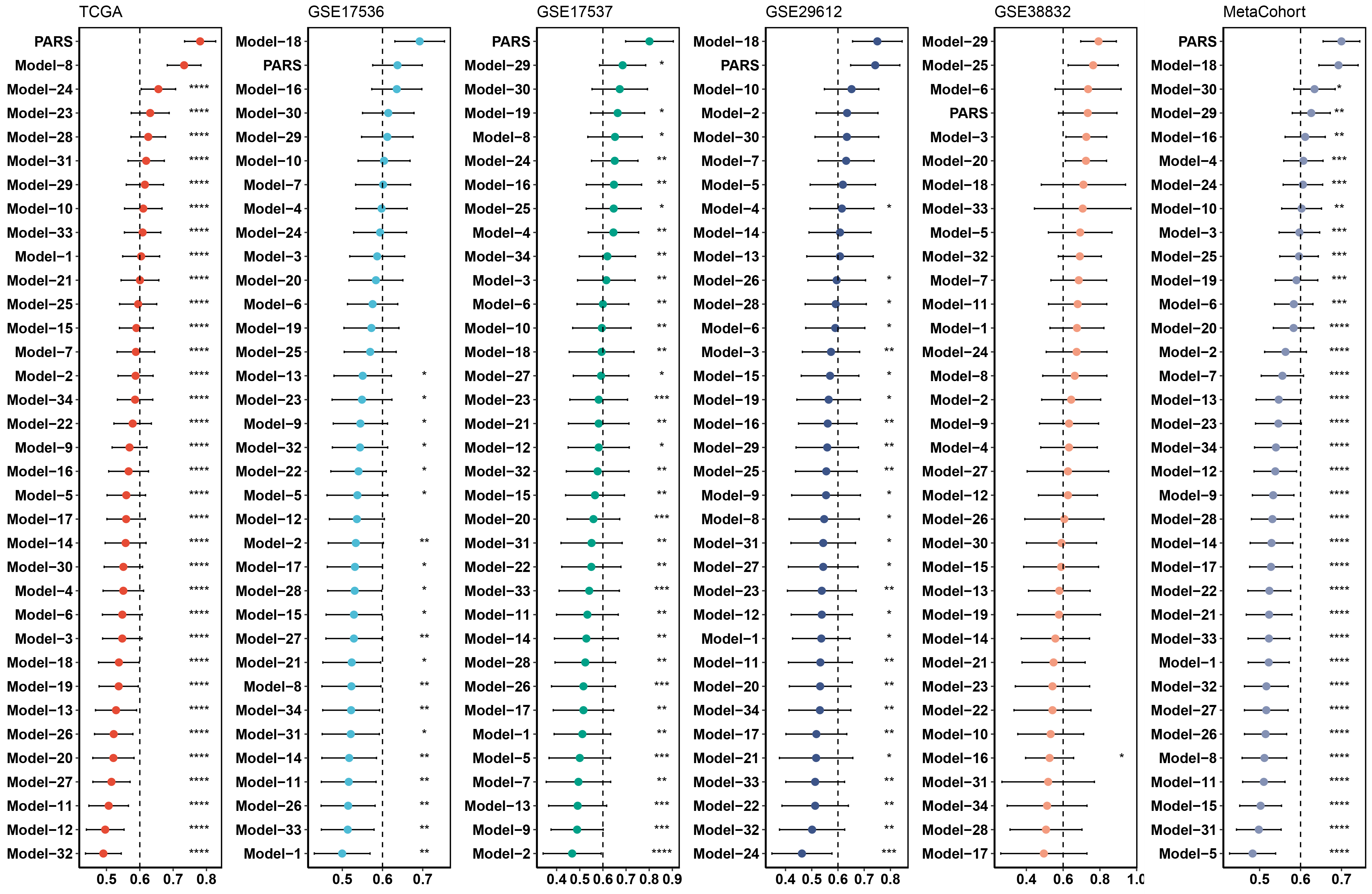


**Fig. S3** Comparisons of the c-index value between PRS and other reported signature in TCGA, GSE17536, GSE17537, GSE29612, GSE38832, and meta cohorts, respectively.


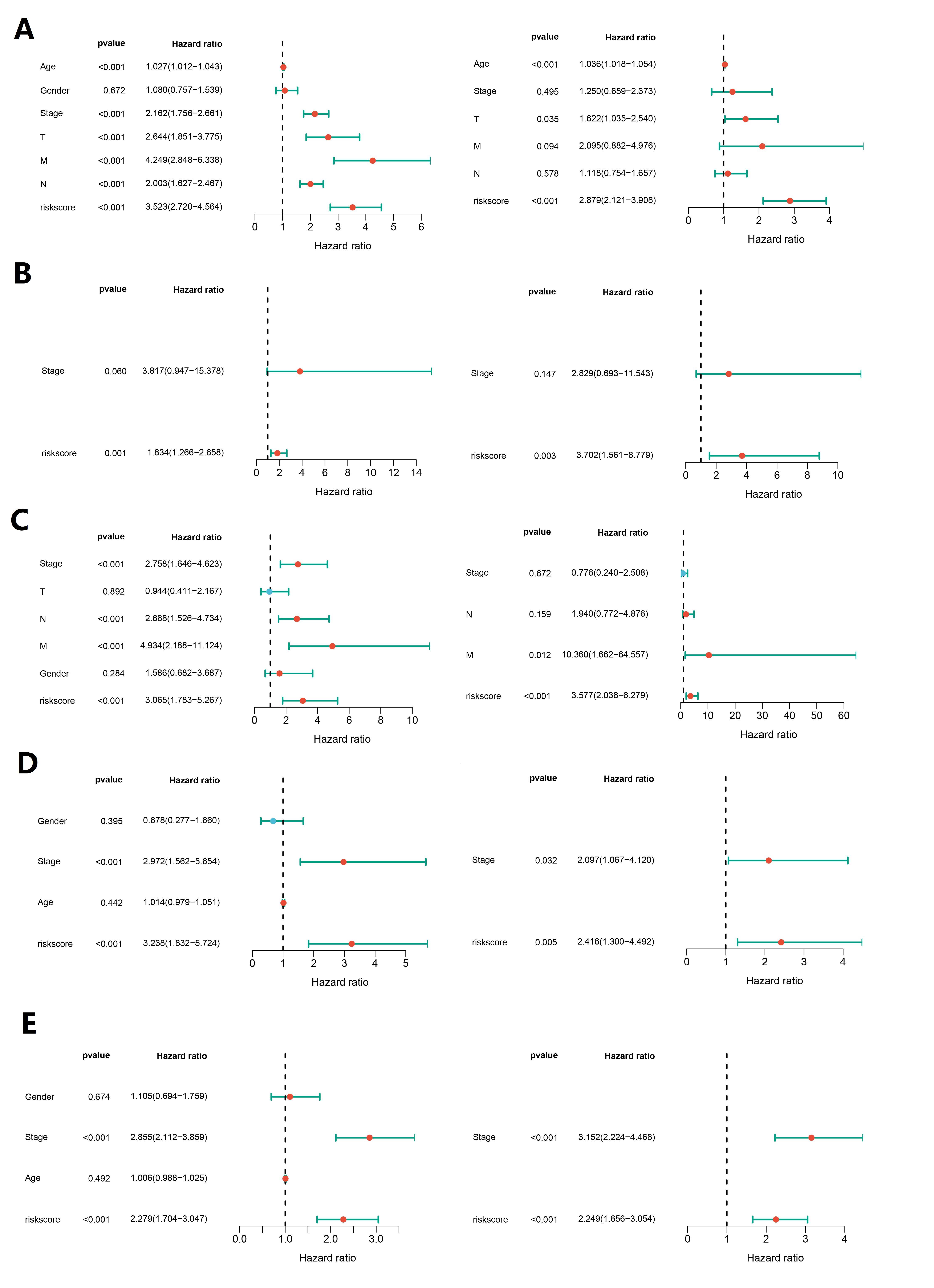


**Fig. S4** Estimation of the prognostic value of PRS and clinical factors. Univariate and multivariate cox regression analysis was performed to identify the independence of PRS in TCGA (A), GSE38832 (B), GSE29612 (C), GSE17537 (D) and GSE29612 (E) cohorts.


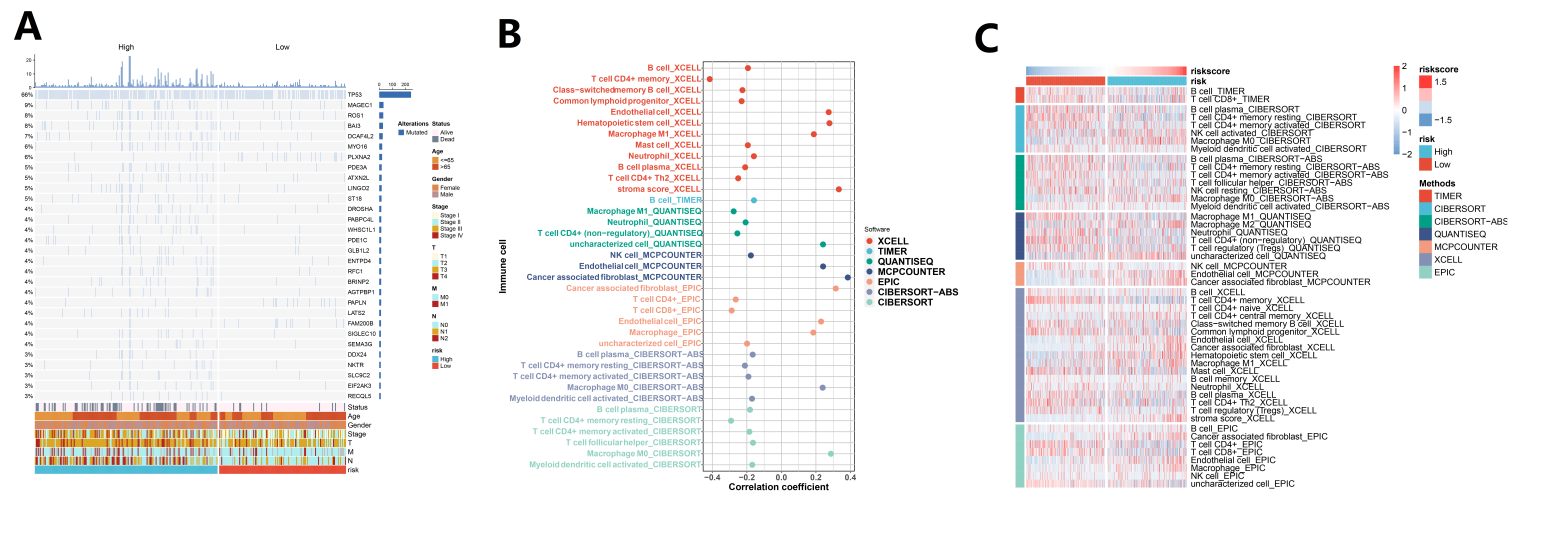


**Fig. S5** The association of PRS group and genomic mutations, immune infiltration. (A) SNP mutation landscape between high and low PRS group. (B-C) A dot plot and heatmap showed the correlation between PRS and immune infiltration cells.


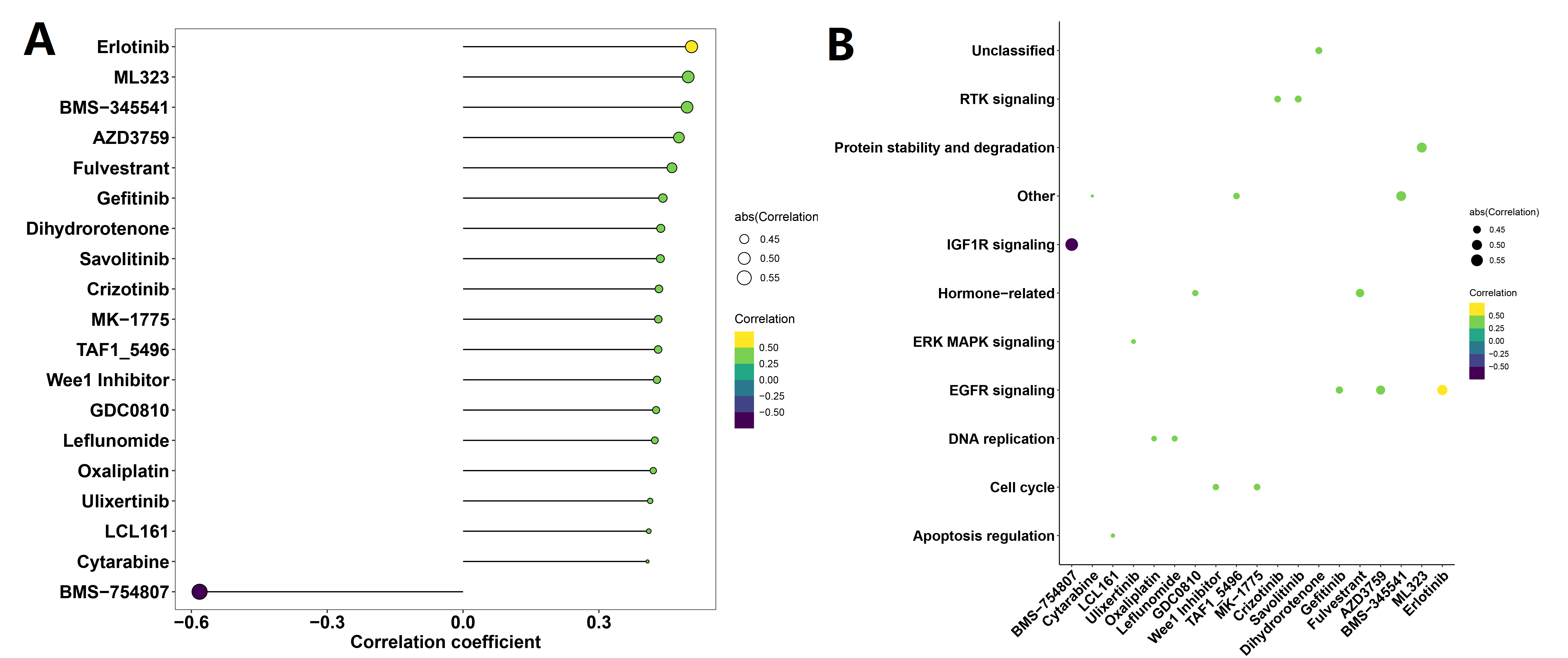


**Fig. S6** Identification of candidate drugs associated with ELN. (A) Correlation analysis between the AUC value of drugs and ELN expression. (B) The molecular mechanism of the correlated drugs.


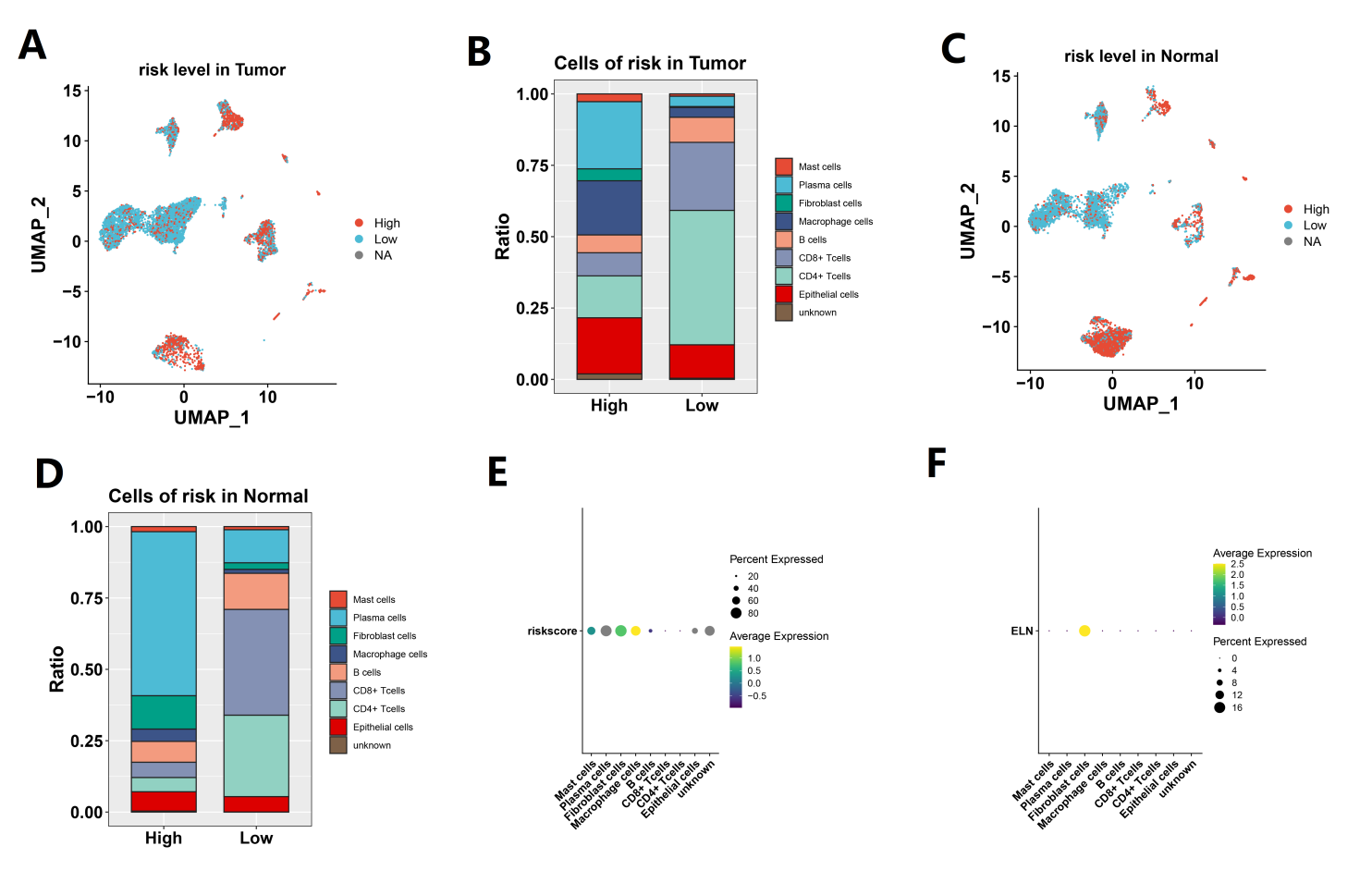


Fig. S7 Single cell RNAseq analysis to evaluate the PRS and ELN level. (A-D) The proportion of cells in high and low PRS group stratify by normal and tumor tissue. (E-F) Scatter plot showed the distribution of PRS score and ELN in annotated cells.

**Table S1** ELN staining in tumor cells and associations with clinicopathologic characteristics

|  | ELN expression | | | | |
| --- | --- | --- | --- | --- | --- |
| Variable | No. | Negative | Positive | χ^2^ | p -value |
| **Age(years)** |  |  |  | 0.286 | 0.593 |
| ＜60 | 60 | 29 | 31 |  |  |
| ≥60 | 71 | 31 | 40 |  |  |
| **Gender** |  |  |  | 0.286 | 0.593 |
| Male | 71 | 31 | 40 |  |  |
| Female | 60 | 29 | 31 |  |  |
| **Location** |  |  |  | 1.218 | 0.270 |
| colon | 63 | 32 | 31 |  |  |
| rectum | 68 | 28 | 40 |  |  |
| **Differentiation** |  |  |  | 4.674 | 0.031* |
| poor | 18 | 4 | 14 |  |  |
| well | 113 | 56 | 57 |  |  |
| **pT stage** |  |  |  | 4.386 | 0.036* |
| T1-T2 | 21 | 14 | 7 |  |  |
| T3-T4 | 110 | 46 | 64 |  |  |
| **pN stage** |  |  |  | 8.990 | 0.003** |
| N0 | 60 | 36 | 24 |  |  |
| N1-N2 | 71 | 24 | 47 |  |  |
| **pM stage** |  |  |  | 5.671 | 0.018* |
| M0 | 109 | 55 | 54 |  |  |
| M1 | 22 | 5 | 17 |  |  |
| **AJCC stage** |  |  |  | 10.725 | 0.001** |
| I | 17 | 13 | 4 |  |  |
| II | 36 | 23 | 13 |  |  |
| III | 56 | 19 | 37 |  |  |
| IV | 22 | 5 | 17 |  |  |
| **CEA** |  |  |  | 0.004 | 0.948 |
| ≤5.0(Negative) | 79 | 36 | 43 |  |  |
| ＞5.0(Positive) | 52 | 24 | 28 |  |  |
| **CA199** |  |  |  | 0.168 | 0.682 |
| ≤37.0(Negative) | 114 | 53 | 61 |  |  |
| >37.0(Positive) | 17 | 7 | 10 |  |  |
| **Total** | 131 | 60 | 71 |  |  |
| Significance level as indicated: *, p<0.05; **, p<0.01; ***, p<0.001. | | | | | |
